# SOX11 is a sensitive and specific marker for pulmonary high-grade neuroendocrine tumors

**DOI:** 10.1186/s13000-021-01186-0

**Published:** 2022-01-07

**Authors:** Lu Yu, Yuting Dong, Jin Xue, Sanpeng Xu, Guoping Wang, Dong Kuang, Yaqi Duan

**Affiliations:** 1 Department of Pathology, Zhongnan Hospital of Wuhan University, Wuhan, 430071 People’s Republic of China; 2 Institute of Pathology, Tongji Hospital, Tongji Medical College, Huazhong University of Science and Technology, 1095 Jiefang Avenue, Wuhan, 430030 People’s Republic of China; 3 Department of Pathology, School of Basic Medical Science, Tongji Medical College, Huazhong University of Science and Technology, Wuhan, 430030 People’s Republic of China

**Keywords:** SOX11, Immunohistochemistry, Small cell lung carcinoma, Large cell neuroendocrine carcinoma

## Abstract

**Background:**

Synaptophysin (SYN), chromogranin A (CGA), CD56 and insulinoma-associated protein 1 (INSM1) are proposed neuroendocrine (NE) markers used for diagnosis of pulmonary NE tumors. These NE markers have been identified in subsets of non-NE tumors requiring differential diagnosis, thus we sought to explore new NE markers.

**Methods:**

We evaluated the immunohistochemical expression of SOX11, a transcription factor involved in neurogenesis, in pulmonary NE tumors and large cell carcinomas (LCCs).

**Results:**

We found that SOX11 showed a sensitivity similar to INSM1 and CGA, and less than SYN and CD56 in small cell lung carcinomas (SCLCs) and large cell neuroendocrine carcinomas (LCNECs). While SOX11 is more specific than the other four markers for diagnosis of high-grade neuroendocrine carcinomas (HG-NECs) because 1) None of LCCs (0/63), the most challenging non-NE tumor type for differential diagnosis due to overlapped morphology with LCNECs displayed SOX11 positivity. While expression of at least one of SYN, CGA, CD56 or INSM1 was identified in approximately 60% (18/30) of LCCs. 2) SOX11 was only expressed in 1 of 37 carcinoid tumors in contrast to diffuse expression of SYN, CGA, CD56 and INSM1. In HG-NECs, we noticed that SOX11 was a good complementary marker for SCLC diagnosis as it was positive in 7 of 18 SYN^−^/CGA^−^/CD56^−^ SCLCs and 3 of 8 SYN^−^/CGA^−^/CD56^−^/INSM1^−^ SCLCs, and SOX11 positivity in 4 of 6 SYN^−^/CGA^−^/CD56^−^ cases previously diagnosed as LCCs with NE morphology provides additional evidence of NE differentiation for reclassification into LCNECs, which was further confirmed by electromicroscopical identification of neurosecretory granules. We also found SOX11 expression cannot predict the prognosis in patients with HG-NECs.

**Conclusions:**

Therefore, SOX11 is a useful complementary transcriptional NE marker for diagnosis and differential diagnosis of SCLC and LCNEC.

## Background

Neuroendocrine (NE) tumors encompass a spectrum of tumors, from low-grade typical carcinoid, intermediate-grade atypical carcinoid to high-grade neuroendocrine carcinoma (HG-NEC), including small cell lung carcinoma (SCLC) and large cell neuroendocrine carcinoma (LCNEC) [1]. Accurate diagnosis of HG-NECs is important as LCNEC and SCLC are clinicopathologically and genetically different from other pulmonary carcinomas with poor survival and limited therapeutic options [1]. Currently the diagnosis is based on the combination of NE morphology and differentiation assessed by immunohistochemistry (IHC) and/or electron microscopy. Synaptophysin (SYN), chromogranin A (CGA) and CD56 are traditionally World Health Organization (WHO) proposed IHC markers used for evaluation in NE differentiation. However, in routine surgical practice some non-NE lung carcinomas, particularly adenocarcinoma and large cell carcinoma (LCC) may show NE differentiation by exhibiting positive staining of NE markers, [2] and a small proportion of SCLCs may not express those markers. Recent years, quite a few other markers have been proposed to indicate NE differentiation including phenotype markers (neuron-specific enolase, NSE [3]; microtubule associated proteins − 2, MAP-2 [4]; histidine decarboxylase, HDC [5]) and NE lineage markers (achaete-scute homolog 1, ASCL1 [6]; and insulinoma-associated protein 1, INSM1 [7]). Only INSM1 shows better sensitivity or/and specificity than SYN, CGA or CD56 [8–10].

The transcription factor *SOX11*, mapping to chromosome 2p25.3, is a member of the *SRY* box-containing (*SOX*) family, which comprises a group of transcription factors that accomplish important tasks during the determination of cell fate [11]. As a member of the C-group of the *SOX* gene family, *SOX11* has been found to operate downstream of proneural bHLH proteins (such as NGN2 and ASCL1), which commit progenitor cells to a neurogenic program to establish a neuronal phenotype. Identification of SOX11 expression in medulloblastoma further confirmed its functional relevance to neurogenesis [12]. In addition, SOX11 has been found to be aberrantly expressed in mantle cell lymphoma, subsets of Burkitt lymphomas, lymphoblastic leukemias and hairy cell leukemias, [13–16] though its role in lymphopoiesis remains unknown yet. Similar to neurogenesis, pulmonary NE cell fate specification during lung development is suggested to be controlled by interplay between bHLH proteins (ASCL1) and Notch target protein HES1 [17]. Using mouse genetics differentiation of pulmonary neuroendocrine cells are found to be depend on INSM1 as mutation of *INSM1* blocks terminal differentiation and expression of phenotype markers by upregulating HES1 protein and interferes with maintenance of ASCL1 expression [18]. While the expression and functional role of SOX11 in pulmonary NE cell differentiation and tumorogenesis are not well studied yet. One recent study revealed that *SOX11* mRNA expression is significantly upregulated in SCLC and LCNEC and absent expression of SOX11 is correlated with prolonged survival [19]. In our recent publication we have already found that a high percentage of HG-NECs, particularly SCLCs, were immunopositive for SOX11 [20]. However, no extensive study comparing the expression of SOX11 with other common NE markers in pulmonary tumors has been reported so far.

Therefore, we immunohistochemically examined SOX11 expression in different types of pulmonary carcinomas. The high and more specific expression of SOX11 observed in SCLC and LCNEC indicates its strong diagnostic value as a complement to the present IHC panel for HG-NECs.

## Materials and methods

### Tumor specimens

This retrospective study included 547 formalin-fixed paraffin embedded (FFPE) samples of pulmonary carcinomas. These cases all achieved the same confidential diagnosis by two pathologists independently according to the 2021 WHO Classification of Tumors of the Lung, Pleura, Thymus and Heart, [1] which included 109 adenocarcinomas, 76 squamous cell carcinomas, 67 LCCs including 61 typical LCCs and 6 SYN^−^/CGA^−^/CD56^−^ LCCs with NE morphology (LCC-NEMs), 25 typical carcinoids, 12 atypical carcinoids, 199 SCLCs and 59 LCNECs (Table 1). For SCLC samples, around 41% (81/199) are small biopsies.

**Table 1:** Expression of neuroendocrine markers in pulmonary neuroendocrine tumors and large cell carcinoma

Tumor Types	Positive/Total, n/N (%)				
	SOX11	INSM1	CD56	SYN	CGA
SCLC	127/199 (64)	59/79 (75) ^NS^	169/191 (88) ^****^	158/193 (82) ^****^	136/193 (70) ^NS^
LCNEC ^original^	23/59 (39)	19/38 (50) ^NS^	44/56 (79) ^****^	48/59 (81) ^****^	36/59 (61) ^*^
LCNEC ^update^	27/63 (43)	21/40 (53) ^NS^	44/60 (73) ^***^	48/63 (76) ^***^	36/63 (57) ^NS^
Typical carcinoid	0/25 (0)	16/22 (73) ^****^	25/25 (100) ^****^	25/25 (100) ^****^	25/25 (100) ^****^
Atypical carcinoid	1/12 (8)	6/11 (55) ^*^	10/10 (100) ^****^	12/12 (100) ^****^	11/11 (100) ^****^
Typical LCC	0/61 (0)	9/28 (32) ^****^	13/49 (27) ^****^	5/53 (9) ^*^	2/52 (4) ^NS^
LCC-NEM	4/6 (67)	2/4 (50) ^NS^	0/6 (0)	0/6 (0)	0/6 (0)

Abbreviations: SCLC, small cell lung carcinoma; LCNEC, large cell neuroendocrine carcinoma; LCC, large cell carcinoma; LCC-NEM, large cell carcinoma with NE morphology; INSM1, Insulinoma-associated protein 1; SYN, synaptophysin; CGA, chromogranin A

^NS, *, **, ***, ****^refers to the *P*-value of comparing the percentage of different kinds of tumors stained positive between SOX11 and INSM1, CD56, SYN, and CGA using the Fisher exact tests was > 0.05, < 0.05, < 0.01, < 0.001 and < 0.0001, respectively. *P* < 0.05 was considered statistically significant

Note: LCNEC ^original^ refer to the original categorization before SOX11 immunostaining and electron microscope were performed. While LCNEC ^update^ refer to the updated categorization when SOX11^+^ LCC-NEM was regrouped into LCNEC

### Immunohistochemistry

Tissue sections including whole slide of the representative block and tissue microarrays were deparaffinized and hydrated, and endogenous peroxidase activity was blocked. Antigen retrieval was achieved using Dako Target Retrieval Solution, High PH (Dako, Produktionsvej, DK; 50×) in a PT Link set at 98 °C for 25 min. Afterwards the tissue sections were incubated with an anti-SOX11 mouse monoclonal antibody (MRQ-58, Cell MARQU, USA), anti-INSM1 (A-8, Santa Cruz Biotechnology, USA), SYN (MRQ-40, Cell MARQU, USA), CGA (DAK-A3, DAKO, USA), CD56 (123C3D5, Cell MARQU, USA) for 30 min at room temperature, and detection was achieved using an enzyme-conjugated polymer complex adapted for automatic stainers from DAKO (Dako, Dako Autostainer, Produktionsvej, DK). Tumor cells of mantle cell lymphoma were used as a positive control. Paratumoral mature mesenchymal or epithelial cells served as negative controls. All immunostains were recorded for intensity of reactivity (0, none; 1+, weak; 2+, moderate; 3+ strong) and percentage of positive neoplastic cells. Positive immunostaining for CD56, SYN and CGA required 10% or more cells with an intensity of at least 2+ on cytoplasmic or membranous localization [21]. For SOX11 and INSM1, at least 1+ nuclear staining in > 10% of tumor cells was considered positive [10].

### Electron microscopy

FFPE sections were deparaffinized and dehydrated with xylene and acetone, then washed in phosphate buffer 0.1 M and post-fixed with 1% osmium tetroxide at 4 °C for 2 h. Subsequently, the sections were dehydrated through a graded ethanol series (70–100%) and propylene oxide before semisaturated in 50% acetone plus 50% epoxy-resin and embedded in neat resin. Ultrathin sections of 50 nm thickness were collected onto 200 mesh copper grids and examined with a transmission electron microscope (HT7800, HITACHI, Japan) after stained with uranyl acetate and lead citrate. Pictures were acquired using a Slow Scan CCD-camera and iTEM software (Olympus, panasonic, Japan).

## Results

### Comparison of sensitivity and specificity of SOX11 with other NE markers in different types of lung carcinomas

As a positive control, SOX11 nuclear staining was identified in 85% (46/54) of mantle cell lymphomas, which is consistent with previous reports [15]. Next we examined the expression of SOX11 in normal lung tissue and different types of lung cancers, which is compared with the expression of INSM1, SYN, CGA and CD56. In paraneoplastic lung tissue, SOX11 expression was not observed in any mature cells, while INSM1, SYN, CGA and CD56 is positive in pulmonary mature NE cells. In lung cancers, SOX11 was almost not observed in adenocarcinomas (1/109) and squamous cell carcinomas (0/76), while INSM1 was observed in 16% (11/69) adenocarcinomas and 18% (4/22) squamous cell carcinomas (not shown in Table 1).

Among pulmonary non-NE carcinomas, due to lack of specific lineage markers, and demonstration of overlapped cellular morphology with LCNEC and/or NE differentiation by expression NE markers, LCC becomes the most challenging non-NE tumor for differential diagnosis with HG-NEC, particularly LCNEC. In addition, as a temporary subtype of LCC, LCC-NEM is readily regrouped into LCNEC with the usage of new NE markers or other technologies for evaluation of NE differentiation in surgical practice. Considering all above, LCCs including typical LCCs and LCC-NEMs are the non-NE carcinomas included in this study.

**Fig. 1:**
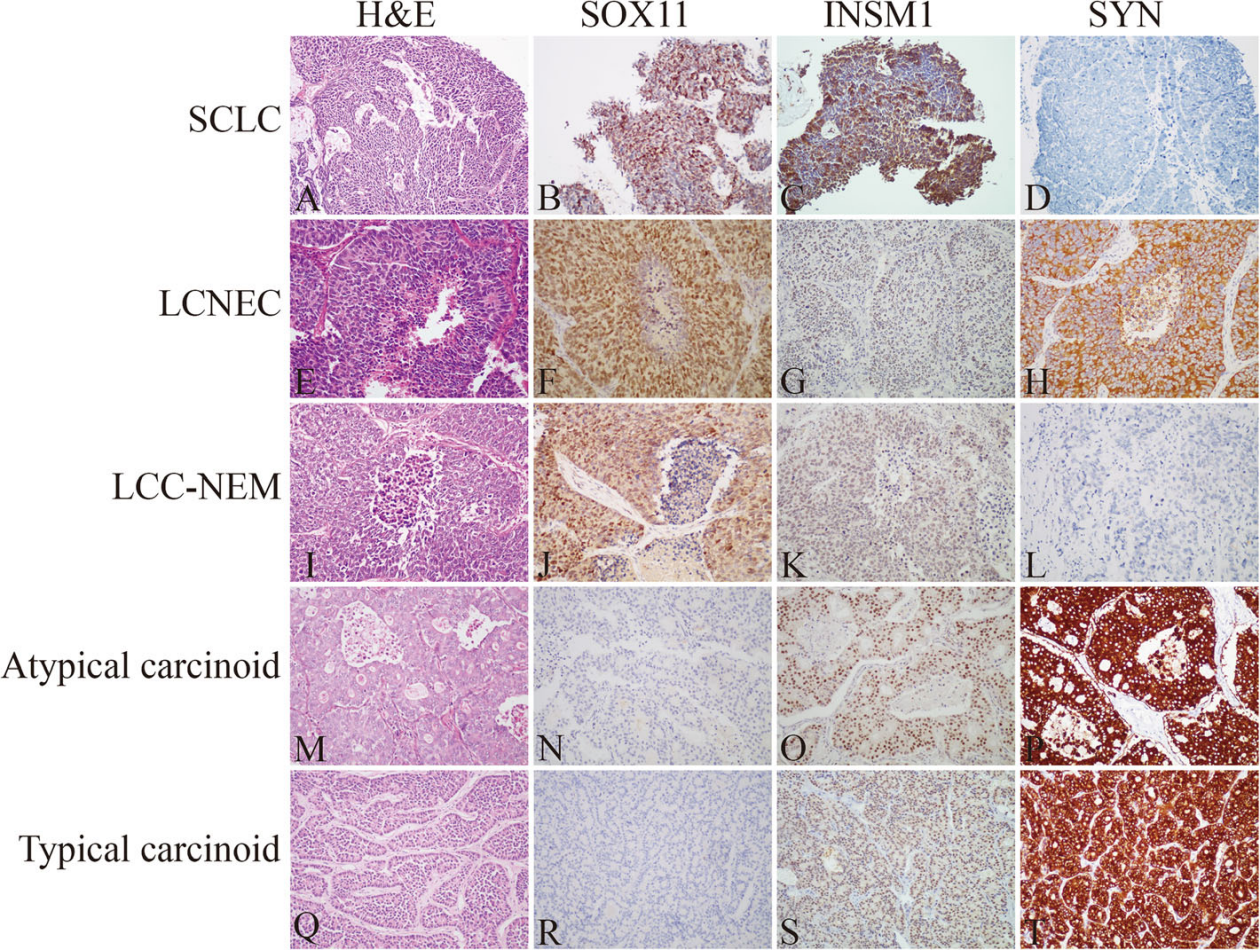
Expression of SOX11, Insulinoma-associated protein 1(INSM1), and synaptophysin (SYN) in small cell lung carcinoma (SCLC), large cell neuroendocrine carcinoma (LCNEC), large cell carcinoma with neuroendocrine morphology (LCC-NEM), atypical carcinoid and typical carcinoid**. A-D**, SCLC. Small densely packed tumor cells exhibit a sheet-like diffuse growth pattern (**A**). Tumor cells demonstrate strong nuclear staining of SOX11 and INSM1(**B, C**), negative staining of SYN (**D**) and other traditional neuroendocrine markers. **E-H**, LCNEC. Rosette-like structures and large zones of necrosis are observed in a LCNEC (**G**). Positive immunohistochemical staining was observed for SOX11, INSM1 and SYN (**F, G, H**). **I-L**, LCC-NEM. Large cell carcinoma demonstrating an organoid growth pattern (**I**). Tumor cells are positive for SOX11 and INSM1 (**J, K**), negative for SYN (**L**) and other traditional neuroendocrine markers. **M- T**, typical and atypical carcinoid. Organoid and rosette structure was observed in an atypical carcinoid (**M**) and typical carcinoid (**Q**). SOX11 expression is not found in the atypical carcinoid (**N**) and typical carcinoid (**R**). INSM1 and SYN reactivity are present in tumour cells of atypical carcinoid (**O, P**) and typical carcinoid (**S, T**). Magnification (**A-I**): 200x

As demonstrated in Fig. 1 and Table 1, the positivity of SOX11 in SCLCs and LCNECs^update^ (4 SOX11^+^ LCC-NEMs were regrouped into LCNECs) were 64% (127/199) and 43% (27/63), that were similar to INSM1 (SCLC: 75%, 59/79, *p* > 0.05; LCNEC: 53%, 21/40, *p* > 0.05) and CGA (SCLC: 70%, 136/193, *p* > 0.05; LCNEC: 57%, 36/63, *p* > 0.05), and less than SYN (SCLC: 82%, 158/193, *p* < 0.0001; LCNEC: 76%, 48/63, *p* < 0.001) and CD56 (SCLC: 88%, 169/191, *p* < 0.0001; LCNEC: 73%, 44/60, *p* < 0.001). For carcinoids, in contrast to diffuse expression of INSM1, SYN, CGA and CD56 in most of the cases, positivity staining of SOX11 was observed in none of typical carcinoids (0/25) and only 1/12 of atypical carcinoids (Table 1). For typical LCCs, INSM1, SYN, CGA and CD56 were identified in 32% (9/28), 9% (5/53), 4% (2/52) and 27% (13/49) of typical LCCs, respectively, and there was 64% of typical LCC cases demonstrating expression of at least 1 of the 4 NE markers. However, different from those markers, SOX11 was not identified in any cases of typical LCCs (0/61).

Based on above findings, among these NE markers, SOX11 shows a good sensitivity comparable with INSM1 and CGA and is more specific for differentiating HG-NECs from LCCs and carinoids (Table 2).

**Table 2:** Sensitivity and Specificity of SOX11 and INSM1

	HG-NEC ^update^ vs. LCC ^update^		HG-NEC ^update^ vs. Carcinoid tumour	
	SOX11 (%)	INSM1 (%)	SOX11 (%)	INSM1 (%)
Sensitivity	59	67	59	67
Specificity	100	70	97	0
Positive Predictive Value	100	90	99	100
Negative Predictive Value	37	35	25	0

Abbreviations: HG-NEC, high-grade neuroendocrine carcinoma; LCC, large cell carcinoma; INSM1, insulinoma-associated protein 1

NOTE: HG-NEC ^update^ and LCC ^update^ refer to the updated categorization when SOX11^+^ LCC with neuroendocrine morphology was regrouped into large cell neuroendocrine carcinoma

Sensitivity = true-positive/(true-positive + false-negative); Specificity = true-negative/(true-negative + false-positive); Positive predictive value = true-positive/(true-positive + false-positive); Negative predictive value = true-negative/(true-negative + false-negative)

### SOX11 is a useful complement for diagnosis of HG-NEC negative for traditional NE markers

Expression of SOX11 as well as INSM1, SYN, CGA and CD56, was further carefully analyzed in SCLC and LCNEC cases. In Table 3, we summarize the frequency of reactivity of 5 markers in SCLCs. Approximately 6% (5/79) of cases were negative for all 5 markers, including SOX11. Among 18 SYN^−^/CGA^−^/CD56^−^ SCLCs diagnosed by typical morphological characteristics, 39% (7/18) cases were positive for SOX11 and 53% (9/17) for INSM1. It is known that SCLC has distinctive morphology and evidence of NE immunophenotype is not necessary for diagnosis. However, when SYN^−^/CGA^−^/CD56^−^ specimens exhibit ambiguous NE structures and cytological features due to crush artifacts, both SOX11 and INSM1 could be useful complement to assistant the diagnosis of SCLC.

**Table 3:** Immunoreactivity for Neuroendocrine Markers in SCLC

Markers	No. of Cases	Positive markers	% of Cases
All negative	5		6
1 positive	9	1: CD56+	11
		3: SOX11+	
		5: INSM1+	
2 positive	6	1: CD56+, SYN+	8
		1: SOX11+, CD56+	
		4: SOX11+, INSM1+	
3 positive	10	1: SOX11+, SYN+, CGA+	13
		1: SOX11+, CD56+, SYN+	
		2: CD56+, SYN+, INSM1+	
		6: CD56+, SYN+, CGA+	
4 positive	20	1: SOX11+, CD56+, SYN+, CGA+	25
		5: SOX11+, CD56+, SYN+, INSM1+	
		14: CD56+, SYN+, CGA+, INSM1+	
All positive	29		37
Total no.	79^a^		

Abbreviations: SCLC, small cell lung carcinoma; INSM1, Insulinoma-associated protein 1; SYN, synaptophysin; CGA, chromogranin A

^a^One hundred and twenty cases were excluded from the 199 cases of SCLC because immunohistochemical staining was not performed for all markers

**Fig. 2:**
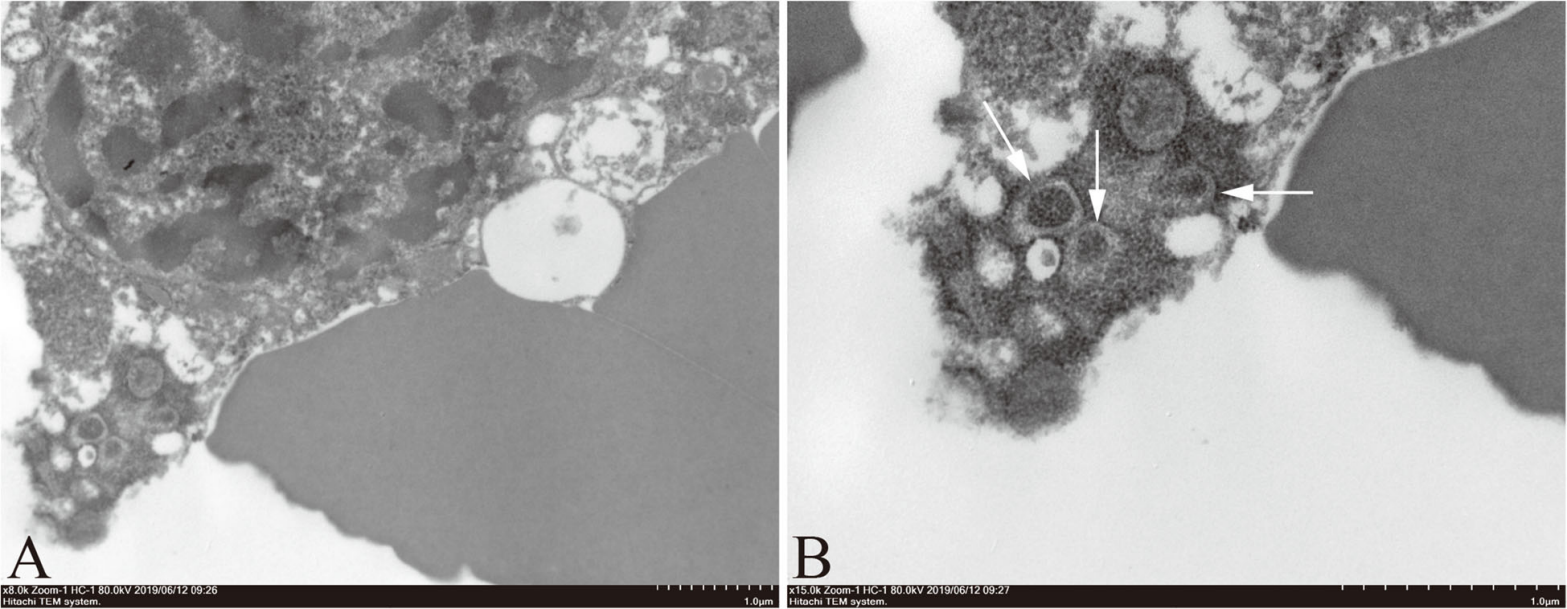
Electron photomicrograph of SOX11^+^ large cell carcinoma with neuroendocrine morphology. **A,** The tumor cell is rich in euchromatin and has distinct nucleolus with large nuclei (uranyl acetate and lead citrate, × 5000). **B,** Cytoplasmic dense-core (neurosecretory) granules surrounded by a transparent envelope (synaptic protein) within the cytoplasm are observed (As indicated by the white arrow) (uranyl acetate and lead citrate, × 15,000)

Different from SCLC, diagnosis of LCNEC requires the evidence of NE differentiation based on IHC or electron microscope. Thus, exploration of new NE markers would result in reclassification of previously diagnosed LCC-NEM into LCNEC. Interestingly we found that SOX11 was identified in 4/6 (67%) of LCC-NEMs in our daily practice due to the absence of SYN, CGA and CD56 expression. Only two cases of 4 SOX11^+^ LCC-NEM were available for INSM1 test and were all positive for INSM1. These four SOX11^+^ LCC-NEM samples were then taken for electron microscopic examination, by which NE granules were observed in two well-preserved FFPE samples (Fig. 2), but failed in another two poorly-preserved samples due to long-term storage. With the further confirmation NE differentiation by the electron microscope, we regrouped these SOX11^+^ LCC-NEMs as LCNECs. Therefore, statistically 13, 26, 16, 21 and 24% of LCNEC^update^ cases showed positive staining for 1, 2, 3, 4 and 5 NE markers, respectively (Table 4).

**Table 4:** Immunoreactivity for Neuroendocrine Markers in LCNEC ^update^

Markers	No. of Cases	Positive markers	% of Cases
All negative	0		0
1 positive	5	1: SYN+	13
		4: CD56+	
2 positive	10	2: SOX11+, INSM1+	26
		2: CD56+, SYN+	
		3: SOX11+, CD56+	
		3: SYN+, CGA+	
3 positive	6	1: SOX11+, SYN+, CGA+	16
		1: CD56+, SYN+, INSM1+	
		4: CD56+, SYN+, CGA+	
4 positive	8	1: SOX11+, CD56+, SYN+, INSM1+	21
		7: CD56+, SYN+, CGA+, INSM1+	
All positive	9		24
Total no.	38^a^		

Abbreviations: LCNEC, large cell neuroendocrine carcinoma; SYN, synaptophysin; INSM1, Insulinoma-associated protein 1; CGA, chromogranin A

^a^Twenty-five cases were excluded from the 63 cases of LCNEC^update^ because immunohistochemical staining was not performed for all markers

Note: LCNEC^update^ refer to the updated categorization when SOX11+ large cell carcinoma with neuroendocrine morphology was regrouped into LCNEC

Taken together, we propose that SOX11 is an useful complementary NE marker for diagnosis of HG-NECs, particularly when they are absence of NE immunophenotype with the traditional NE markers (SYN, CGA and CD56).

### Influence of the SOX11 expression on overall survival in SCLCs and LCNECs

**Fig. 3:**
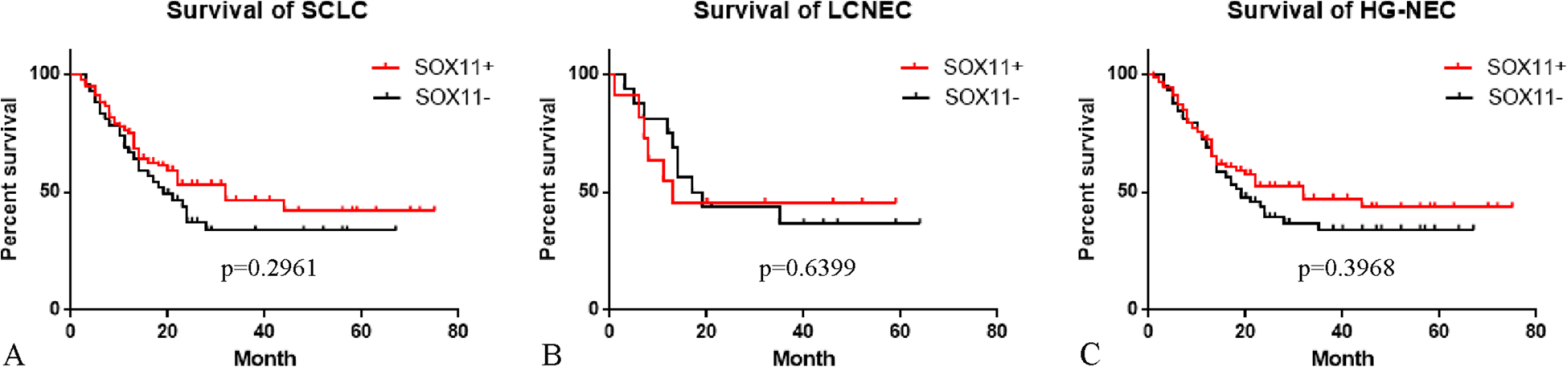
The overall survival of small cell lung carcinomas (SCLCs), large cell neuroendocrine carcinomas (LCNECs) and high-grade neuroendocrine carcinomas (HG-NECs) between SOX11 positive and negative cases. Kaplan-Meier survival curve showing that SOX11 exhibit no impact on SCLCs, LCNECs and HG-NECs patient survival (**A**, **B** and **C**, *p* = 0.2961, 0.6399 and 0.3968, respectively)

In addition to diagnosis, we also evaluated the prognostic role of SOX11 in SCLCs, LCNECs and all HG-NECs. Overall survival (OS) rates were estimated by Kaplan-Meier survival curves, and we found that there is no significantly difference of OS between SOX11 positive and negative cases in SCLCs, LCNECs or HG-NECs (*p* = 0.2961, 0.6399 and 0.3968, respectively, Fig. 3).

## Discussion

Currently, the WHO classification of NE tumors is based on morphological features in combination with the evidence of NE differentiation from IHC and/or electron microscope. Past studies have shown that SYN, CGA, CD56 and INSM1 are reliable NE markers to detect NE differentiation, and widely applied in daily surgical practice. However, some non-NE lung cancers show equivocal immunoreactivity for these markers, [22] and some HG-NECs may not express them [23]. Thus, continued studies are needed to identify additional NE biomarkers. Our previous publication revealed that transcriptional factor SOX11 downstream of ASCL1 is expressed in high percentages of HG-NECs, particularly SCLCs [20]. In this study, we further compared SOX11 expression with other NE markers in different kinds of pulmonary carcinomas to access its diagnostic value, and found that SOX11 is a sensitive and more specific NE marker and can be a complement for diagnosis of HG-NECs.

SYN, CGA and CD56 are traditional NE phenotype markers first used for diagnosis of SCLC and LCNEC. Later researchers have developed a few other NE markers that include phenotype markers NSE, MAP-2 and HDC and the transcriptional lineage-determining marker ASCL1 and INSM1. NSE is an enolase present in neurons and neuroendocrine cells indicating tumors derived from these cell types. NSE is not used in clinical practice as it cannot differentiate between different subtypes of NETs and is significantly elevated in poorly differentiated tumors, which offers no particular benefit over CgA [24]. MAP-2 and HDC are NE markers discovered in the last 10 years. They show similar sensitivity and specificity with SYN and CD56 for pulmonary NE tumors. However, as phenotype markers similar to SYN, CGA and CD56, they cannot provide good additive value on the basis of present NE panel [4].

In recent years, transcriptional factors regulating NE cell differentiation are well studied. It is known that in the mouse lung, reduced NOTCH pathway activity via INSM1 induced suppression of HES1 promotes NE differentiation by upregulating ASCL1 in ASCL1^+^ NE- Clara cell progenitors [18, 25]. However, different from stable transcriptional expression of INSM1 during NE cell differentiation of embryonic development, ASCL1 play an important role at early stage of differentiation [6]. Correspondingly in NE tumor studies, ASCL1 and INSM1 exhibit different expression patterns. ASCL1 is preferentially observed in pulmonary HG-NECs, less frequently in carcinoid tumors, [7] while INSM1 is constantly expressed in all pulmonary NE tumors with even higher sensitivity in carcinoid. For diagnosis of HG-NECs, INSM1 seems to show better performance as ASCL1 shows less sensitivity (92 and 68% in SCLCs and LCNECs for INSM1; 63 and 59% in SCLCs and LCNECs for ASCL1), and similar specificity as compared with INSM1 (1–17% in NSCLCs for INSM1 and around 10% in NSCLCs for ASCL1) [6–10, 25].

During neurogenesis, *SOX11* functions downstream of proneural bHLH proteins (ASCL1 et al) as a critical activator that promotes precursor cells to further differentiate and adopt a neuronal phenotype [26]. Though up to now SOX11 has not been found to be the downstream of ASCL1 in pulmonary NE cell differentiation, NE differentiation of prostate cancer cells is suggested to be partially mediated by SOX11, [27] and a recent paper shows that *SOX11* mRNA is upregulated in pulmonary HG-NECs [19]. Therefore we compared the sensitivity and specificity of SOX11 with SYN, CGA, CD56 and INSM1 in pulmonary NE and non-NE tumors. As reference markers, SYN, CGA and CD56 showed similar sensitivities and specificities with the other reports [21, 28–30]. While INSM1 showed a relatively lower sensitivity and specificity than the other reports, [7–10] which might be due to different experiment conditions. In this study SOX11 is identified in 64% of SCLCs and 43% of LCNECs demonstrating sensitivities similar to INSM1 and CGA (Fig. 1, Table 1). Beneficially, SOX11 hardly detected in NSCLCs, particularly LCCs, and carcinoids, exhibiting a significantly better specificity than all the other 4 markers in the diagnosis of HG-NECs (Table 2). The high specificity of SOX11 helps in distinguishing SCLCs and LCNECs from NSCLCs with NE differentiation and carcinoid tumors. Such differential diagnosis is important as majority of the studies indicate that NE differentiation of NSCLCs has no impact on the patient survival and are not prognostic factors, and support that NSCLCs with NE feature follow the same therapeutic strategy of NSCLCs without NE feature, [31] though controversial opinion has been reported [22, 32].

For SCLCs, the diagnosis is primarily based on histological features characterized by sheets/organoid /nests pattern, scant cytoplasm, a small nucleus, fine granular nuclear chromatin and inconspicuous nucleoli [1]. Immunohistochemical demonstration of NE differentiation is not necessary. In fact, around 10% of SCLC cases lack SYN, CGA and CD56 expression [23]. However, in practice, ambiguous morphology due to crush artifacts is not rare and positivity of SOX11 in 7 of 18 SYN^−^/CGA^−^/CD56^−^ SCLCs and 3 of 8 SYN^−^/CGA^−^/CD56^−^/INSM1^−^ SCLCs provide evidence on the confidential diagnosis of SCLC. For LCNECs, NE differentiation demonstrated by SOX11 positivity in 4 out of 6 LCC-NEMs and neurosecretory granules by electron microscope make us reclassify the SOX11^+^ LCC-NEM into LCNECs. Such reclassification is reasonable because LCC-NEMs based on the absence of SYN, CGA and CD56 expression has an associated poor prognosis similar to LCNECs [33]. In summary, SOX11 is a useful complementary NE marker for diagnosis of both SCLCs and LCNECs, especially SYN^−^/CGA^−^/CD56^−^/INSM1^−^ SCLCs and LCNECs.

The prognostic value of SOX11 expression has been studied in many different kinds of tumors. Literatures revealed the ambiguous role of SOX11 in predicting tumours prognosis, which is particularly dependent on cancer types. In MCLs, several studies discussed the prognostic role of SOX11, while the conclusion was still controversial yet. In gastric cancer, [34] astrocytic gliomas [35] and high-grade epithelial ovarian cancers, [36] increased expression of SOX11 is associated with better prognosis, while in breast cancer [37] and cutaneous malignant melanoma, [38] with poor prognosis. Recently a study of 15 SCLC patients showed that elevated *SOX11* mRNA expression is correlated with poor outcome [19]. However our larger cohort study showed that there is no significant difference of overall survival between SOX11^+^ and SOX11^−^ cases in 199 SCLCs or 63 LCNECs or all HG-NECs (Fig. 3).

## Conclusions

In conclusion, SOX11, a transcriptional factor related to neurogenesis, is a sensitive and more specific NE marker than SYN, CGA, CD56 and INSM1 for the diagnosis of pulmonary HG-NECs. SOX11 constitutes an important complement to the present panel of NE markers to distinguish HG-NECs from carcinoid and non-NE cancers, especially LCCs. The functional relevance between SOX11 and ASCL1 in NE differentiation remains to be further studied.

## Data Availability

All data generated or analysed during this study are included in this published article.

## Abbreviations

NE: neuroendocrine
HG-NEC: high-grade neuroendocrine carcinoma
SCLC: small cell lung carcinoma
LCNEC: large cell neuroendocrine carcinoma
IHC: immunohistochemistry
SYN: synaptophysin
CGA: chromogranin A
WHO: World Health Organization
LCC: large cell carcinoma
NSE: neuron-specific enolase
MAP-2: microtubule associated proteins − 2
HDC: histidine decarboxylase
ASCL1: achaete-scute homolog 1
INSM1: Insulinoma-associated protein 1
SOX: SRY box containing
FFPE: formalin-fixed paraffin embedded
LCC-NEM: large cell carcinoma with NE morphology
OS: Overall survival
